# A semi-empirical relation based on temperature difference and filling ratio in a closed loop pulsating heat pipe: A numerical study

**DOI:** 10.1371/journal.pone.0309108

**Published:** 2024-11-19

**Authors:** Vasanth Balamurugan, Shahid Mian P., Md. Jahid Hasan, Natteri M. Sudharsan

**Affiliations:** 1 Department of Mechanical Engineering, Rajalakshmi Engineering College, Chennai, Tamil Nadu, India; 2 Department of Mechanical and Production Engineering (MPE), Islamic University of Technology (IUT), Board Bazar, Gazipur, Bangladesh; GH Raisoni College of Engineering and Management Pune, INDIA

## Abstract

A closed-loop pulsating heat pipe (CLPHP) is an attractive passive cooling system for electronic components. The design of CLPHP is challenging due to the complex nature of thermo-hydrodynamic coupling. This study investigates the heat transfer efficiency of a CLPHP using water as the working fluid. The heat transfer rate is evaluated for a volume fraction of 0.3-0.7 and an evaporator temperature of 323-373 K. From the computed results, a regression analysis is performed to generate a semi-empirical equation. The empirical relation for heat transfer rate (Q) as a function of the temperature difference and filling ratio was found to match the CFD results. Similarly, a semi-empirical equation for heat flux (q) as a function of non-dimensionless numbers is presented to calculate the heat transfer rate (Q) for various filling ratios, and found to match CFD results. A force plot measuring the net force acting on the slugs is presented for various filling ratios and evaporator temperatures. The net force plot will help optimize the design of the CLPHP and improve its efficiency. When comparing slug formation pulsatile cycle and thermal efficiency, 0.5 volume fraction was found to be optimum. For this filling ratio (0.5) heat transfer rate is enhanced from 40% to 86% when the evaporator temperature is increased by 15%.

## 1. Introduction

The increase in cloud storage has created huge data centers that require massive cooling loads. Also, the miniaturization of electronic chips leads to high Watt loading (W/m^2^). Approximately 55% of electronic component failures are primarily caused by high operating temperatures [1]. Cooling is one way to prevent failures by incorporating heat sinks and forced cooling. However, passive cooling is now commonly used, as it has no moving parts and is not prone to failure. Passive heat pipes are classified as Flat Plate Pulsating Heat Pipes (FPPHP) and Capillary Tube Pulsating Heat Pipes (CTPHP). Anand Takawale *et al*. [2] studied the performance of CTPHP and FPPHP. They found that CTPHP has better thermal performance than FPPHP since lateral conduction in the FPPHP was found to negatively impact the oscillations of the slug plug. Due to its high efficiency, the tube configuration is one of the most commonly used passive cooling systems. This Closed Loop Pulsating Heat Pipe (CLPHP) configuration has been studied in the past three decades, primarily focusing on improving thermal performance by varying the number of turns [3], tube diameter, tilt angle, filling ratio, and working fluid. The energy transfer happens by extracting heat from the evaporator and transferring it to the condenser through the pulsating motion of alternate vapour plugs and liquid slugs. The diameter is one of the critical factors that help in slug formation and movement. The surface tension forces dominate over gravitational force, and the slugs are stable if the heat pipe diameter is lesser than the critical value of Bo ≤ 2. Gravity significantly affects CLPHP dynamics but is found to be efficient if the system is within 60 to 90 degrees.

However, Bo>2 can lead to bubble formation, which is sufficient for heat transfer to take place and the bond number of water is 0.8 [4]. The latent heat is inversely proportional to the expansion rate of bubbles created in the heater region.


$$Bo=\left(\frac{g(\rho_{l}-\rho_{v})}{\sigma }\right)*{D_{crit}}^{2}$$ (1)

The filling ratio and temperature difference are given preference for study due to the impact on slug formation. Khandekar *et al*. [4] and Piyanun Charoensawan *et al*. [5] developed a semi-empirical model for CLPHP as the understanding of the system is very complex due to thermo-hydrodynamic solid coupling. Their model was used to estimate the heat flux considering dimensionless parameters. Himel Barua *et al*. [6] presented that the most suitable filling ratio for water as working fluid was found to be in the range of 25-80%, and the most efficient filling ratio was found to be 50%, wherein the evaporation rate and condensation rate were close attaining stability. Qu and Wang [7] performed an experimental study on a CLPHP. They found the thermal performance was better for a 2 and 2.5mm ID with water as the working fluid. It was observed from their thermal resistance plot that the temperature difference between the evaporator and condenser for a filling ratio of 40-60% for an input power of 15-127 W varied from 10K to 40K, respectively. The reason for the change in thermal resistance due to the filling ratio was not investigated, but a 40% filling ratio was observed to be optimum. A similar observation of around 50% filling ratio with water as working fluid was found to be comparatively efficient [8–15]. All these studies were experimental, and no clear understanding of the effect of thermo-hydrodynamics was presented. It is observed that heat removal happens due to the pulsating motion of water slugs carrying heat from the evaporator to the condenser in a loop. Several factors are in play for the extraction of pool heat. It is felt that the role of water alone can be studied from the point of heat removal due to factors such as the formation of vapor plugs and liquid slugs, which are, in turn, due to temperature and pressure inside the system, which indirectly influences the capillary forces and surface tension. The effect of surface tension on the heat transfer coefficient and thermal resistance has been studied by varying the surface tension of the fluid by adopting various strategies such as using surfactants or blending of high carbon alcohols and varying the thermal characteristics using nanoparticles [16–25].

While the increase in viscosity constrained the fluid’s highest velocity within PHP’s capillary tube structure, the impact of surface tension outweighed this limitation. In light of this, using a surfactant solution, PHP performs better at larger filling ratios and higher heat loads. The surface tension of the fluid is a function of temperature and contact angle that dictates the stability of the water slug [16]. The combined impact of surface tension and viscosity on the thermal performance of PHP was studied using a computational technique involving various liquids, indicating the likely cause for improved performance of PHP by adding different surfactants [17]. The performance was enhanced by doping the working fluid with nanoparticles or adding miscible liquids like alcohols such as ethanol, butanol, and acetone [18–20]. This indicates that thermal performance has been enhanced through decreased surface tension. Pure alcohols lower the surface tension to extreme values, causing a premature dry-out situation and increasing phase shift, adversely affecting PHP performance. For this reason, alcohol is added to water in minimal quantity rather than as a neat working fluid.

Nanoparticles have proved to be an essential factor in enhancing thermal performance. The experimental procedures consider a volume fraction of less than 1% of nanoparticles. Nanofluids are an innovative form of heat transfer fluids showing great potential to enhance the basic thermo physical attributes of traditional coolants in thermal management devices like cooling units for electronic components [21–24]. Hybrid nanofluids are binary fluids with nanoparticles. Zufar *et al*. [25] studied the role of hybrid nanofluids in multi-turn closed loop pulsating pipe, and a 2D model was developed. Further, it was found that at all filling ratios, hybrid nanofluids showed quicker start-up pulsations and required less heating power for start-up than water. It was observed that dry-out tends to occur when the filling ratio is low. The nanofluids outperformed pure water in terms of thermal performance.

Due to the expensive experimental set-up, visualization tools, and inability to measure specific parameters in experiments, CFD is preferred as an alternative method of study. Numerical simulations help to evaluate the heat transfer characteristics of a heat pipe at the lowest possible cost before conducting actual experiments [26–32]. The use of 3D simulations has been limited due to the high computational time and cost. However, a 3D fluid flow simulation is necessary to visualize the liquid slug geometry better. Wang *et al*. [27] performed a 3D CFD simulation to evaluate the impacts of surface wettability on the thermal efficiency of a single-loop pulsating heat pipe.

Two-dimensional (2D) simulation of photovoltaic cooling in a single loop of pulsating heat pipe (PHP) was studied, and the effect of PHP on cooling was found to be effective [28]. The chaotic flow in a closed loop pulsating heat pipe was explored by providing heat flux and constant temperature boundary conditions to the evaporator and condenser using 2D simulations. Furthermore, the effects of geometry and multisource heat input on the velocity flow direction and heat flux were studied using simulations [32]. The experimental work in open literature implemented either a constant heat flux or constant evaporator temperature as input. In reality, the processor’s temperature needs to be below a specific value, and the flux extracted will vary with time depending on the nature of the operation. Hence, simulating the effectiveness of CLPHP using constant wall temperature seems appropriate. A non-linear regression model has been used to experimentally estimate the thermal resistance of the nanofluid with various filling ratios and condenser temperatures [33]. Further, ANN models have been built to calculate thermal resistance by considering the geometric parameters and dimensionless numbers obtained by conducting experiments on a PHP [34].

Recently, Reagen *et al*. [35] studied the flow reversals in an annular chamber with water as a working fluid, the prediction was carried out using the ET Kalman filter, and it was found that CFD coupled with predictions helped in better prediction accuracies. The properties of heat exchanger characteristics were studied under bridge deck heating conditions. The simulation predicted that heat exchangers buried underground with ethylene performed better [36].

More recently, the performance of CLPHP was enhanced using the Taguchi method and ANOVA by considering various parameters. It was found that heat input has a huge impact on the performance, followed by the inclination angle of CLPHP and filling ratio [37]. Shi *et al*. [38] studied the effect of the evaporator-to-condenser ratio on the operating characteristic of PHP and reported that heat flux and length ratio have a maximum impact on starting characteristics of PHP.

Heat removal in a CLPHP is a complex phenomenon due to thermo-hydrodynamic interactions requiring semi-empirical formulation or data analysis using regression modelling, ANN, etc. However, the main disadvantage of the previous models is that they do not consider the system’s dynamics and can only predict the behaviour in a particular range, which limits the ability to capture the thermo-hydrodynamic response. A predictive model considering the temperature difference between the evaporator and condenser and the filling ratio of CLPHP has not been developed. The filling ratio is critical, and there is a need to optimize the filling ratio for a wide range of evaporator temperatures. The numerical simulations have not considered the effect of temperature on surface tension, which directly affects the heat transfer rate. The present work incorporates a User-defined function (UDF) that would alter the surface tension (hydrodynamic) with respect to temperature changes inside the system (thermodynamic) and the pressure with respect to temperature. The net force acting on the slugs due to thermo-hydrodynamic coupling is analyzed to get a good understating of the working of CLPHP. A regression model was developed to predict that the semi-empirical formulation would be more precise than the model presented in the open literature. Therefore, the present study provides a better understanding of the thermo-hydrodynamic coupling. This understanding and semi-empirical model can be utilized in industrial electronics cooling and to increase the efficiency of the electronic components. CLPHP is also used in solar panel cooling and space control applications.

## 2. Computational methodology

### 2.1 Governing equations

Computational Fluid Dynamics (CFD) was carried out using ANSYS and the governing and mathematical equations are described below:

Continuity equation:

$$\frac{\partial ({\rho u}_{i})}{{\partial x}_{i}}=0$$ (2)

Momentum Equation:

$$\frac{\partial ({\rho u}_{i})}{\partial t}+\frac{\partial ({\rho u}_{i}u_{j})}{\partial x_{j}}=\frac{\partial \rho }{\partial x_{i}}+\frac{\partial }{\partial x_{j}}\left(\mu \frac{\partial (u_{i})}{\partial x_{j}}.\bar{\rho {u\prime }_{i}{u\prime }_{j}}\right)$$ (3)

Energy Equation:

$$\frac{\partial }{\partial x_{i}}(\rho T)+\frac{\partial }{\partial x_{i}}(\rho u_{i}T)=\frac{\partial }{\partial x_{i}}\left(\frac{\gamma }{C_{p}}\frac{\partial T}{\partial x_{i}}\right)$$ (4)

The turbulence model was chosen to account for the unstable flow and the equations are described below:

$$\frac{\partial }{\partial t}(\rho k)+\frac{\partial }{\partial x_{i}}(\rho ku_{i})=\frac{\partial x}{\partial x_{j}}\left[(\mu +\frac{\mu_{t}}{\sigma_{k}})\frac{\partial k}{\partial x_{j}}\right]+G_{k}+G_{b}-\rho \varepsilon -Y_{M}-S_{k}$$ (5)

$$\frac{\partial }{\partial t}(\rho \varepsilon )+\frac{\partial }{\partial x_{i}}(\rho \varepsilon u_{i})=\frac{\partial }{\partial x_{j}}\left[(\mu +\frac{\mu_{t}}{\sigma_{\varepsilon }})\frac{\partial \varepsilon }{\partial x_{j}}\right]+C_{1\varepsilon }\frac{\varepsilon }{K}(G_{k}+C_{-\varepsilon }G_{b})-C_{2\varepsilon \rho }{\frac{\varepsilon }{k}}^{2}+S\varepsilon$$ (6)

Where *u*_*i*_ (m/s) is the velocity component and *μ*_*t*_ is the eddy viscosity.

The Volume of fluid (VOF) model simulates the two-phase flow; the implicit scheme is utilized for the time Discretization, and the implicit body force is enabled to consider the pressure gradient. The VOF method considers the two phases to be distinct.

$$\frac{\partial }{\partial x}(\alpha_{v}\rho_{v})+\nabla .(\alpha_{v}\rho_{v}{\vec{v}}_{v})=S_{\alpha_{v}}+\sum_{l=1}^{n}({\dot{m}}_{lv}-{\dot{m}}_{vl})$$ (7)

Where, ${\dot{m}}_{lv}$ and ${\dot{m}}_{vl}$ are mass transfer rates from the liquid phase to the vapour phase and the vapour phase to the liquid phase, respectively. *ρ*_*v*_ is the density of the vapor phase and ${\vec{v}}_{v}$ is the velocity vector of the vapour phase. The vapour phase volume fraction is computed by

$$\sum_{v=1}^{n}\alpha_{v}=1$$ (8)

$$\frac{\alpha_{v}^{n+1}\rho_{v}^{n+1}-\alpha_{v}^{n}\rho_{v}^{n}}{\Delta t}+\sum_{f}p_{v}^{n+1}U_{f}^{n+1}\alpha_{v,f}^{n+1}=[S_{\alpha_{v}}+\sum_{l=1}^{n}({\dot{m}}_{lv}-{\dot{m}}_{vl})]V$$ (9)

The mass transfer between the phases is described as follows:

Evaporation:

$${\dot{m}}_{lv}=\varphi_{e}.\alpha_{l}\rho_{l}\frac{\left(T-T_{Sat}\right)}{T_{Sat}},T>T_{Sat}$$ (10)

Condensation:

$${\dot{m}}_{vl}=\varphi_{c}.\alpha_{v}\rho_{v}\frac{\left(T-T_{Sat}\right)}{T_{Sat}},T<T_{Sat}$$ (11)

Where, *φ*_*c*_ is the relaxation factor used to interpret the interface temperature between the two phases with maximum accuracy, the intermolecular forces of attraction between the liquid particles cause surface tension variations, which is the predominant force and has a significant effect compared to gravitational force in smaller diameters. The Continuum Surface Force (CSF) has been used to model the impact of surface tension.


$$F_{vol}=\sigma_{lv}\frac{\alpha_{l}\rho_{l}k_{v}{\nabla \alpha }_{v}+\alpha_{v}\rho_{v}k_{l}{\nabla \alpha }_{l}}{\frac{1}{2}(\rho_{l}+\rho_{v})}$$ (12)

$$F_{st}=\sigma_{k}\nabla \alpha_{l}$$ (13)

### 2.2 Mathematical equations

The force acting on the water slugs due to the difference in surface tension between the vapour and liquid phases called the Capillary force, has been calculated from Eq 14.


$$F_{cap}=2\pi \cdot r_{i}\cdot \sigma \cdot (cos\;\alpha_{front}-cos\alpha_{back})|dynamic$$ (14)

$$F_{push}=\Delta p.A$$ (15)

Here, *Δp* is the pressure difference between the vapour slug’s back and front [4].


$$Q_{cap}=\frac{(\frac{2\sigma }{r_{eff}})-\rho_{l}gl_{eff}sin\theta }{\begin{array}{c} \frac{32\mu_{\nu }l_{eff}}{d_{\nu }^{2}A_{\nu }\rho_{\nu }h_{fg}} \end{array}}$$ (16)

$$△P_{\nu }=\frac{48\;\mu_{\nu }l_{eff}}{\rho_{\nu }d_{\nu }^{2}r_{\nu }^{2}h_{fg}(1+s^{2})(1-0.63s\;\tan\left(\frac{\pi }{2s}\right))}Q_{cap}$$ (17)

The △*P*_*v*_ of Eqs 16 and 17 were calculated for the water in a liquid state at room temperature. The evaporator pressure increases over time due to the increasing randomness, which increases saturation temperature that would alter the forces causing the oscillations.

The pressure difference is caused by the difference in surface tension induced by temperature. The saturation is temperature, usually a constant value, leading to an overestimation of the pressure inside the CLPHP. To compensate for this, the current study uses a User-Defined Function (UDF) to vary the saturation temperature depending on the vapour pressure. Surface tension is a temperature-dependent variable at every moment, as presented in Eqs 18 and 19.

UDF for temperature as a function of pressure

$$T_{Sat}=\left(\frac{1723.6425}{\left(8.05573-log10(P_{V}/133.322)\right)}-233.08\right)+273.15$$ (18)

UDF for surface tension as a function of temperature

$$\sigma =0.0764-0.0002.T-1\times 10^{-7}.T^{2}$$ (19)

### 2.3 Fluid domain and considered cases

**Fig 1:**
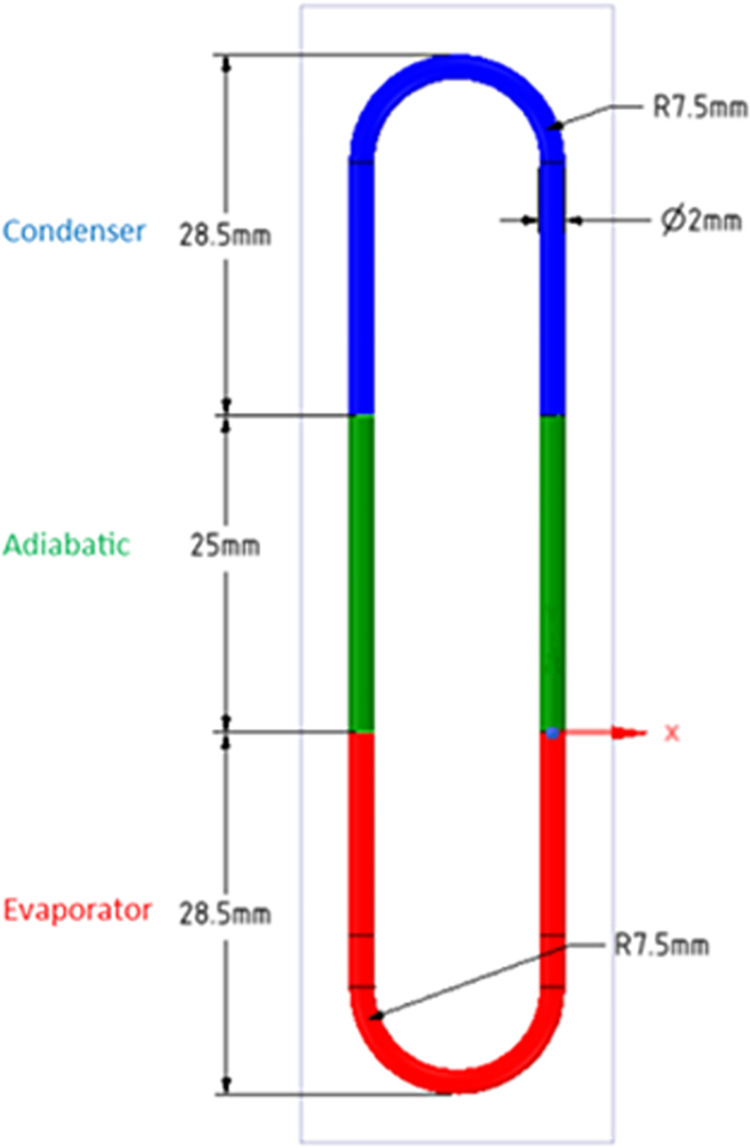
Geometry of single-turn CLPHP.

The computation is done by modelling a single-loop pulsating heat pipe of 2mm diameter, as in **Fig 1**. A three-dimensional (3D) single-turn and double-turn CLPHP simulation model was designed using Ansys Design Modeller. The total length of the CLPHP is 178 mm, and the diameter is 2mm, which is less than the specified Bond Number for water as a working fluid. This aids in the proper functioning of heat pipes. The PHP geometry was divided into three sections, i.e., evaporator, adiabatic, and condenser- section, having a length of 28.5mm, 25 mm, and 28.5mm, respectively, with a loop diameter of 15mm. The evaporator region has a volume of 200mm^3^.

Three filling ratios of 0.3, 0.5, and 0.7 operating at wall temperature of 323K, 348K, and 373K were investigated. The condenser temperature is maintained at 296K for all cases. Thus, the understanding based on constant heat flux (variable wall temperature) would be different from the proposed approach of fixing the wall temperature and estimating the heat extracted from the processor, which is, in effect, the heat input to the evaporator. **Table 1** summarises the 9 cases studied. In the next section, the numerical model and solution methodology is discussed.

**Table 1:** Considered cases for this study.

Case	Filling ratio(γ)	Evaporator temperature (T_evap_) [K]
**Case 1**	0.7	373
**Case 2**	0.7	348
**Case 3**	0.7	323
**Case 4**	0.5	373
**Case 5**	0.5	348
**Case 6**	0.5	323
**Case 7**	0.3	373
**Case 8**	0.3	348
**Case 9**	0.3	323

## 3. Simulation methodology

Simulations were conducted using the finite volume approach. A steady-state simulation was conducted using the CFD software ANSYS FLUENT, employing the pressure-based coupled solver (SIMPLE, SIMPLER, SIMPLEC, PISO). The SIMPLE approach, which is based on pressure-velocity coupling, was selected for the simulation because of its ability to tackle a wide range of heat transfer and fluid flow issues. A second-order strategy was utilized to solve the pressure scheme. The gravitational force and the implicit body force are activated. There are various mass transfer models used in multiphase flow and the most commonly used is the source terms due to mass transfer which does not account for the phase change due to temperature. The cavitation model is used to estimate the mass transfer in two-phase flow however it cannot be accommodated with the VOF model due to its surface tracking scheme. Thus, the Lee model is the best-suited model to estimate the evaporation-condensation phase changes. The Lee model can account for the mass transfer between the water vapour and liquid phases due to condensation and evaporation; the values of 0.1 frequencies are used in the coefficients [3]. The momentum and energy equations presented as Eqs 3 and 4 are discretized using the first-order upwind approach. The laminar flow model was used before to mimic the slug-flow pattern [13]. However, this model did not account for the flow instabilities during the start-up stage. Thus, the *k*−*ε* turbulence model is considered to account for the unstable flow during the initial stage of slug formation due to its high accuracy. The simulation time step is set sufficiently small (10^-5^ seconds) to capture the minute movement of the working fluid. The non-dimensional residual for mass is set as 10^−4^ and 10^-6^ for both the velocity and energy equations. The relaxation factors are set to their default values: pressure = 0.3, density = 1, body forces = 1, momentum = 0.7, vapourization mass = 1, and energy = 1. The simulations were performed on a CPU unit with an Intel(R) CoreTM i7-6700 CPU running at 3.40 GHz and 64 GB of RAM.

### 3.1. Grid independence test

**Fig 2:**
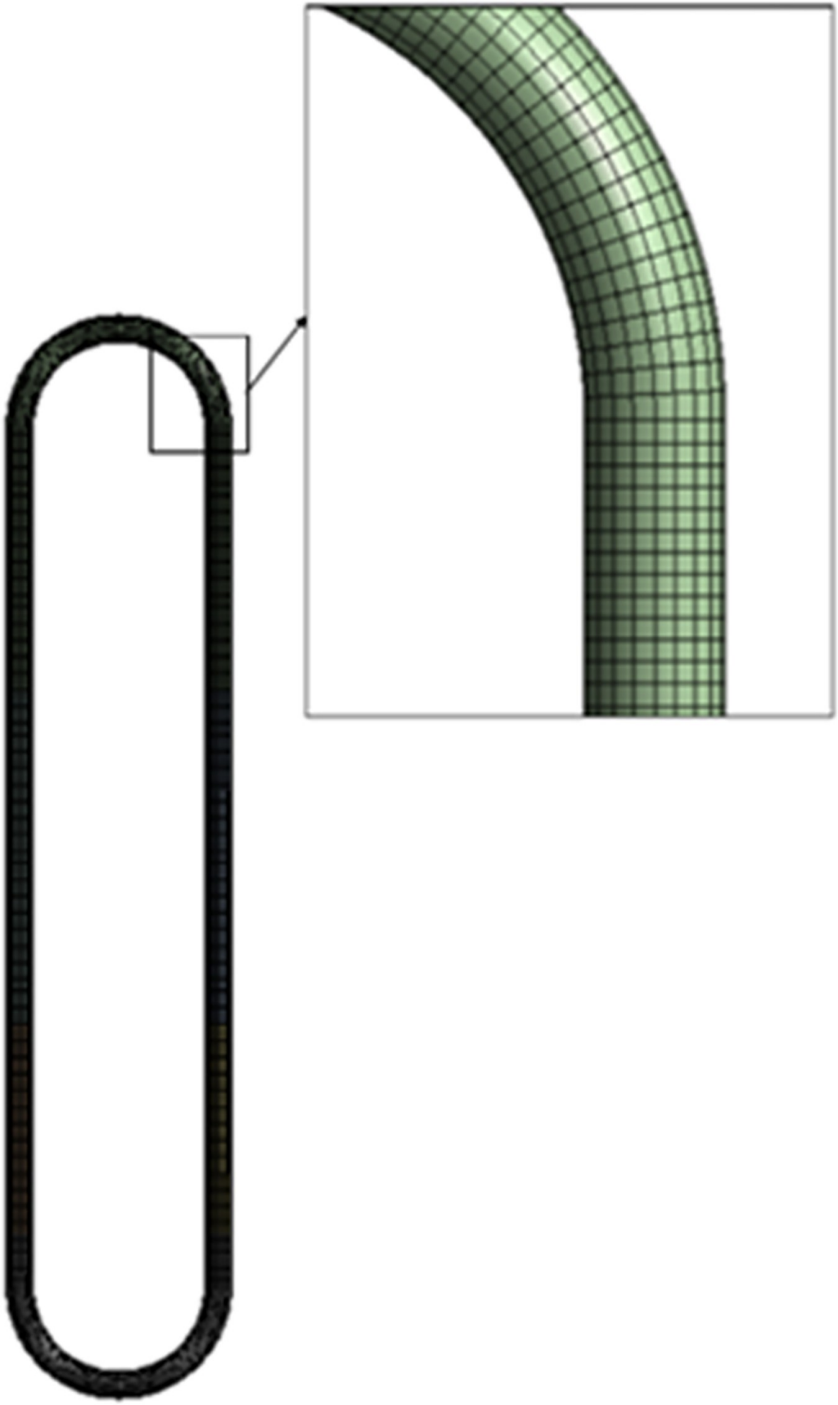
Mesh grids of CLPHP.

**Fig 3:**
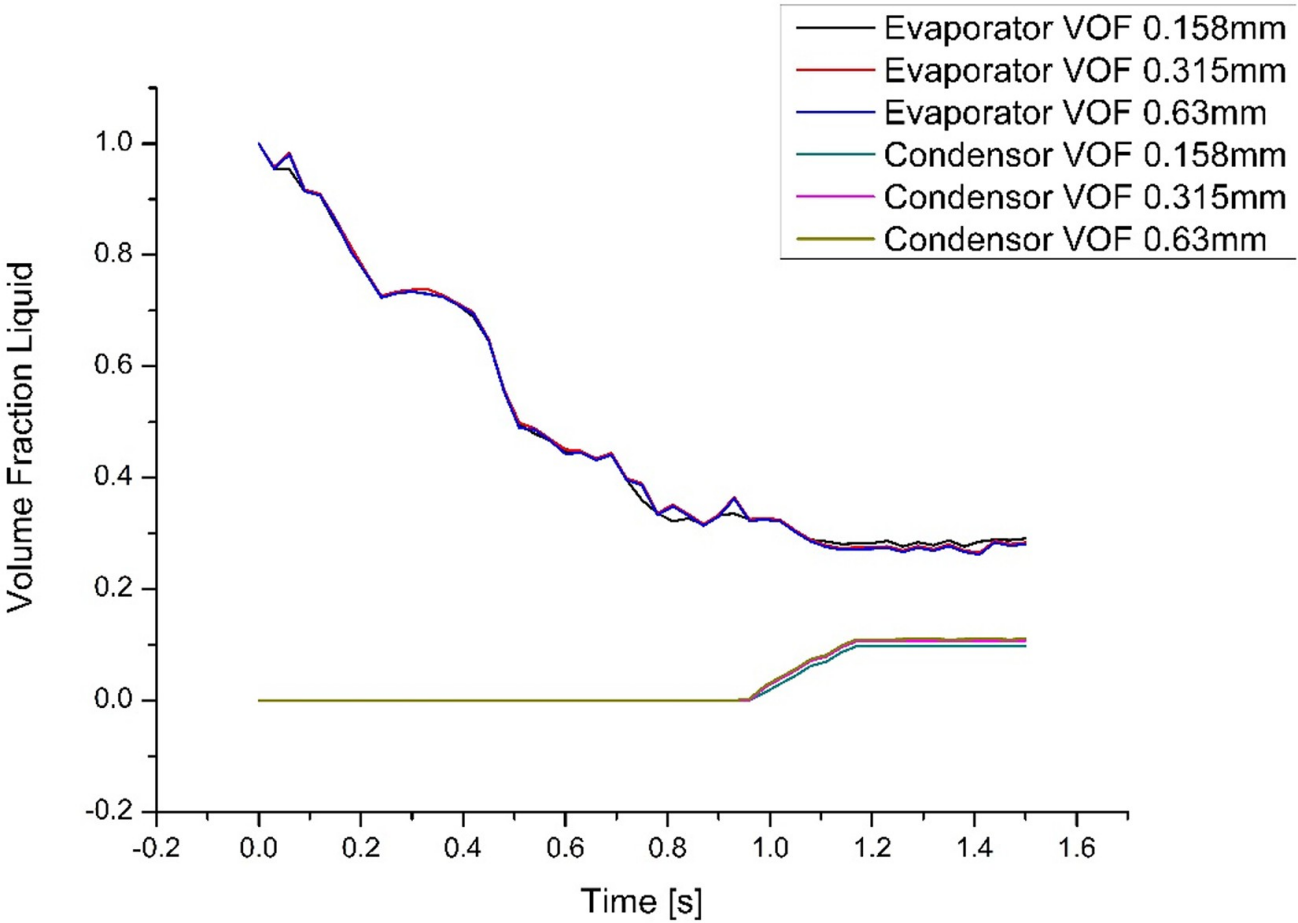
Mesh-independent study of case 7.

The structured hexa-grid mesh with continuous pipe-axis segmentation (**Fig 2**) is used to capture the intricate details of the surface tension. Case 7 is chosen as it has the lowest filling ratio with the highest temperature difference. The mesh sensitivity study for this case consisted of 11436 elements with a size of 0.63mm, 22786 elements with a size of 0.315mm, and 45154 elements with a size of 0.158mm. As visualization of the vapour plugs is of utmost importance, the 0.315 mm element size was chosen within reasonable computational time. The 0.315mm mesh size had an average element quality of 0.8976 and found to be the most suitable for estimating surface tension. The average skewness factor of the mesh is 0.27109 and the average orthogonal quality of the mesh is 0.94052. **Fig 3** presents the VOF for the three grid numbers; deviations were discovered to be at a minimum for case 7.

### 3.2 Boundary conditions

To study the effect of water as the working fluid, the saturation pressure was set to 298K, and the corresponding saturation pressure was set at 3141.51 Pa. The phase change from vapour to liquid state occurs below this temperature. Considering these conditions, the condenser is cooled by convection at 296K, and the evaporator temperatures is maintained above 298K. The adiabatic section is set to steady zero heat flux. The liquid and vapour properties have been specified in **Table 2**. The surface tension of water at 298K is 72 mN/m, and the surface tension was set as a function of saturation temperature in the liquid-vapour state. The saturation temperature is a function of vapour pressure.

**Table 2:** Properties of working fluid.

Properties	Water (Liquid)	Water (Vapour)
Density (Kg m^-3^)	998.2	0.5542
Specific heat (J.Kg^-1^. K^-1^)	4182	2014
Thermal conductivity (W.m^-1^.K^-1^)	0.6	0.0261
Viscosity (Kg.m^-1^.s^-1^)	0.001003	1.34×10^-5^

### 3.3 Validation

**Fig 4:**
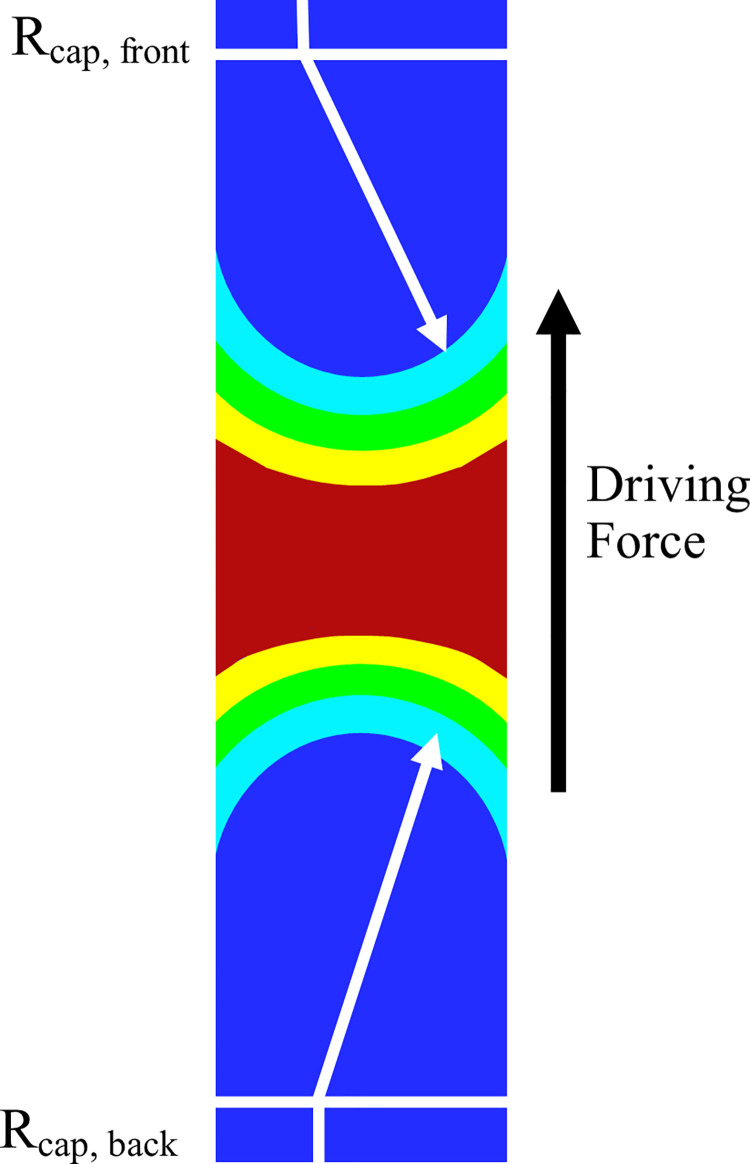
Capillary resistance due to contact angle (CFD).

The current study’s numerical findings were confirmed by comparing them with experimental correlations from other published studies. The net force acting on the slug for Case 6 is validated against a theoretical and experimental correlation by Khandekar *et al*. [4], which is presented in **Fig 4.** As seen in the image, Eqs 14 and 15 are used to compute the forces.

*F*_*cap*_ = 2π · 0.002 · 0.072 (cos (32) – cos (68))

*F*_*cap*,*water*_ = -4.288 × 10^-4^ N

*F*_*push*_ = (3196 – 3155). (1.25 × 10^-4^)

*F*_*push*,*vapor*_ = 51.52 × 10^-4^ N

*F*_*net*_ = *F*_*push*,*vapor*_+*F*_*cap*,*water*_ = 0.864 × 10^-4^ N

*F*_*push*_>*F*_*cap*_ is the cause of the movement of slugs towards the condenser.

The forces are computed using Eqs 14 and 15. The net force obtained above also confirms the force plot obtained over time **Fig 8** thus reinforcing the validity of the results. The equation for pressure difference for a PHP with a wick structure was given by Radaev [40]. This equation was modified for the present study without a wick and given in Eqs 16 and 17. The pressure is also extracted from the CFD results using probe points inside and outside the slug **Fig 4**. The pressure difference from Eqs 16 and 17 was estimated to be 41 Pa, and the value from the simulation results was 42.373 Pa. This validates the current effort.

## 4. Results and discussions

The pulsatile motion depends on the strength of the velocity of the slugs and the corresponding volume fraction plots depicting the movement of slugs (vapour/liquid) is first presented. The force variations acting on the slugs were then analyzed for the varying filling ratios and evaporator temperatures. Effects of thermo-hydrodynamic coupling have been illustrated, showing the force plots over a period. The force plot is a combined effect of capillary force, gravitational force and thermodynamic forces. To determine heat transfer correlations with an extensive application range, it is convenient to express critical thermophysical parameters for heat transfer as non-dimensional values [4].

### 4.1 Water volume fractions for different cases

**Fig 5:**
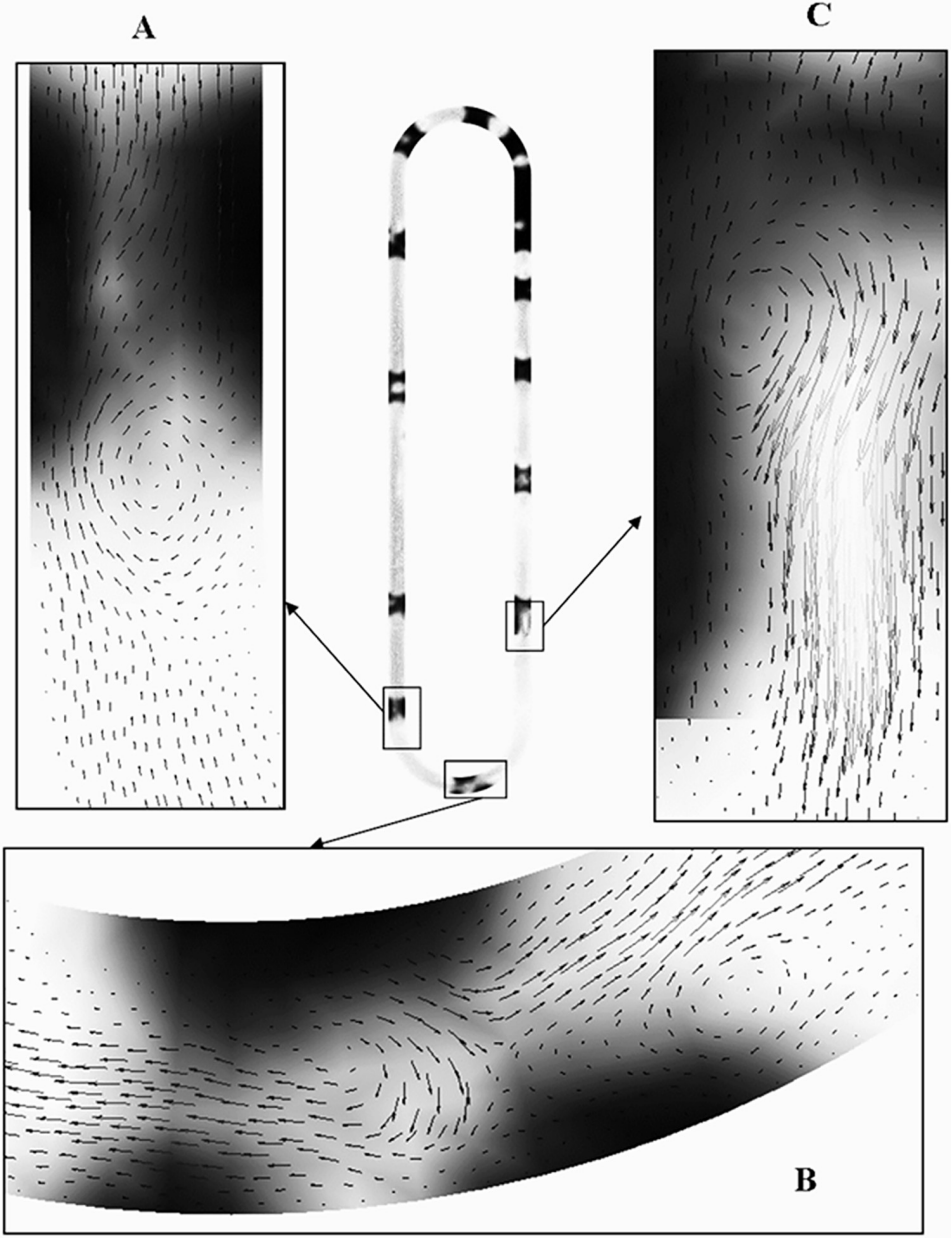
Velocity vector of case 1 at t=1.32s.

The energy transfer in a CLPHP from the evaporator to the condenser is due to the movement of vapour plugs and liquid slugs. The size and pulsating velocity depend on the heating power. With high loading rates and low heating input power, it is characterized by unstable operation due to Intermittent boiling [39]. The velocity vector plot for Case 1 at 1.32s is shown in **Fig 5**. Darker tinge represents the liquid phase. Three regions are marked as A, B and C. The zoomed-in views are also presented. We can observe the formation of vortices and movement along the walls. The formation of vortex is due to the temperature difference and is pronounced at the liquid-vapour interface. This phenomenon is applicable to all cases, but its strength depends upon the volume fraction and temperature gradient. The volume fraction plots for the cases studied are presented and discussed.

**Fig 6:**
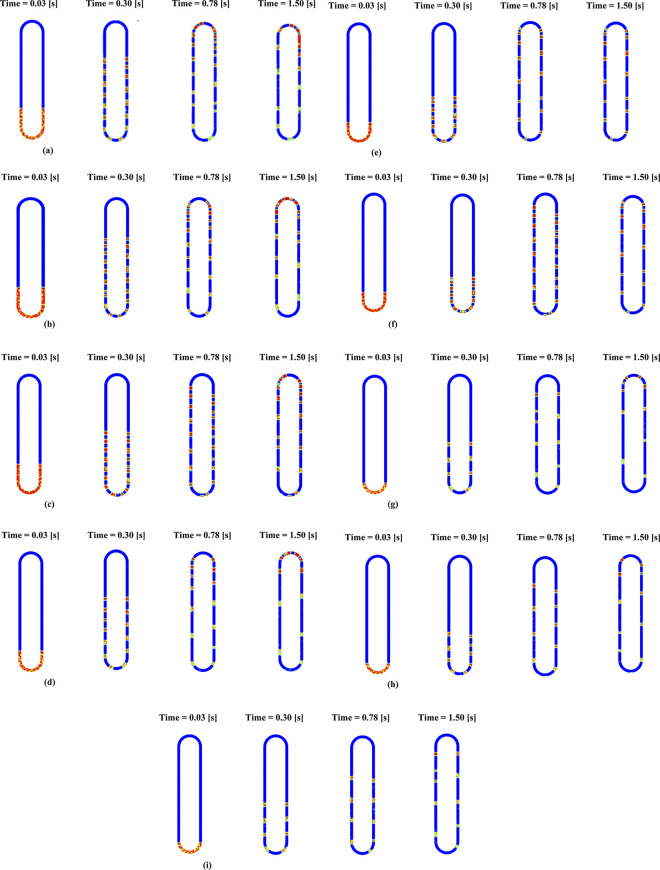
**(a)**: Volume of fraction contour for Case 1 (Fr = 0.7 evap _temp_ = 373 [K]), **(b)**: Volume of fraction contour for Case 2 (Fr = 0.7 evap _temp_ = 348 [K]), **(c)**: Volume of fraction contour for Case 3 (Fr = 0.7 evap _temp_ = 323 [K]), **(d)**: Volume of fraction contour for Case 4 (Fr = 0.5 evap _temp_ = 373 [K]), **(e)**: Volume of fraction contour for Case 5 (Fr = 0.5 evap _temp_ = 348 [K]), **(f)**: Volume of fraction contour for Case 6 (Fr = 0.5 evap _temp_ = 323 [K]), **(g)**: Volume of fraction contour for Case 7 (Fr = 0.3 evap _temp_ = 373 [K]), **(h)**: Volume of fraction contour for Case 8 (Fr = 0.3 evap _temp_ = 348 [K]), **(i)**: Volume of fraction contour for Case 9 (Fr = 0.3 evap _temp_ = 323 [K]).

From the Fig 6(A)–6(I) following are the observations:

In the evaporator, the rate of vaporization and subsequent movement of the slugs increases with temperature. Correspondingly, there is an increase in liquid fraction in the condenser section. It is seen that the rate of increase of liquid in the condenser section is proportional to the volume fraction. At higher volume fractions, the slugs pulsate through the heat pipe, helping better heat transfer; however, at very low volume fractions, there is an inordinate delay for the vapour to reach the condenser and the condensate to flow back. Thus, this is the least efficient.

**Fig 7:**
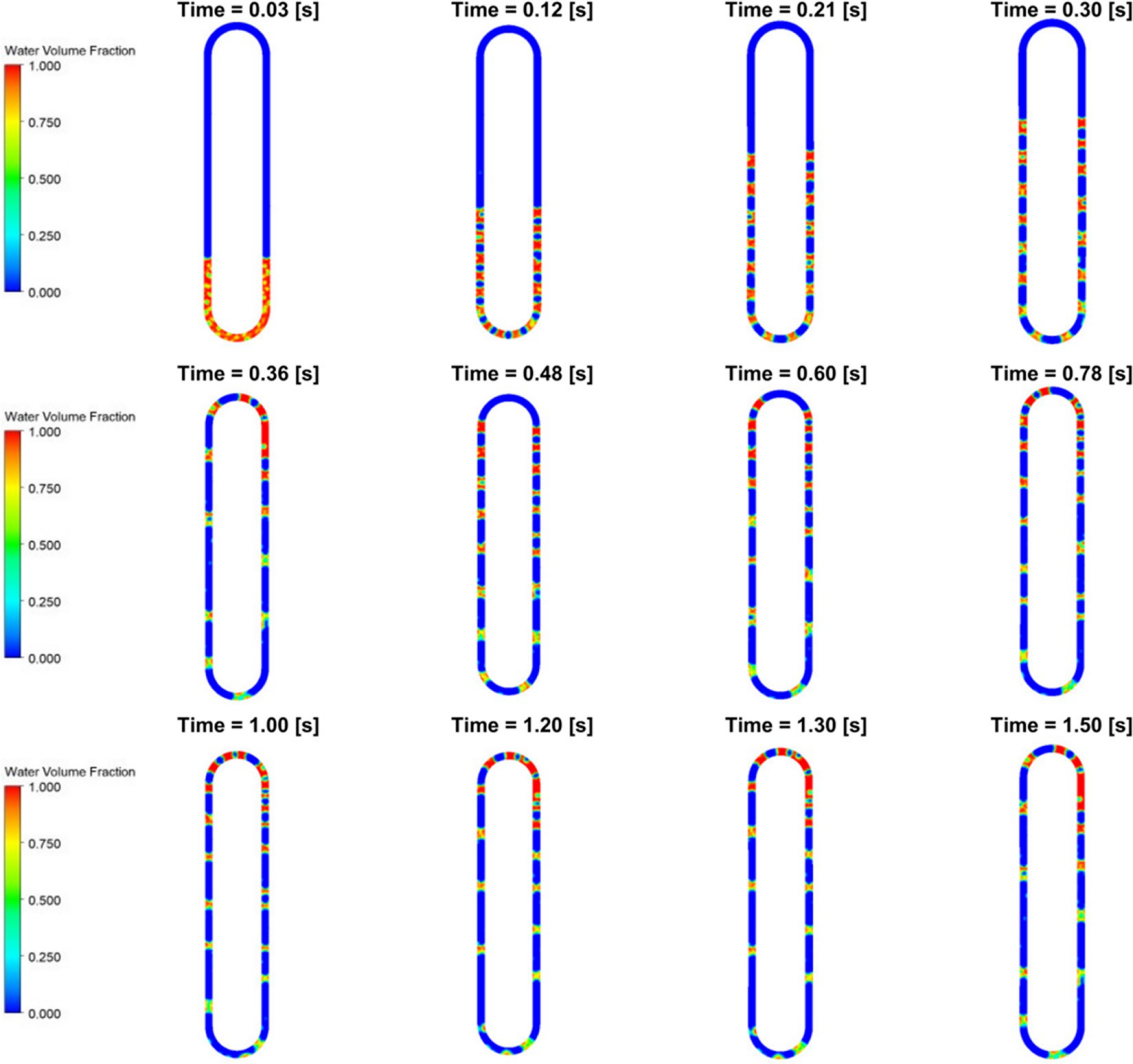
VOF contours at various time instants for case 1.

Here, the 0.7 filling ratio with 373K evaporator wall temperature case is analyzed to understand the flow instability. The case with the highest filling ratio and highest evaporator temperature is analyzed since the effect of temperature and filling ratio is directly related to heat transfer efficiency. **Fig 7** presents the liquid volume fraction in the CLPHP at various time instants for the 0.7 filling ratio with 373K (Case 1). The wall of the evaporator region is heated at a constant temperature, and the liquid present in the evaporator region starts nucleated boiling, creating small-sized bubbles (t = 0.03s). After the expansion, the bubbles increase in size while interacting with the neighbouring smaller bubbles before combining and forming two distinctive regions of the vapour and water phase called the vapour plugs and liquid slugs, respectively (t = 0.12s). The cause of the expansion of vapour plugs was found to be the formation of thin film. The water slugs rise to the adiabatic section and then to the condenser section, creating alternative regions of water slugs and vapour plugs (t = 0.21s and t = 0.30s). The vapour plugs rise upward due to the positive pressure difference above and underneath the plug, which leads to force exertion on the water slugs, leading to the bisected slugs (t = 0.30s). As the water temperature increases, the plugs are distorted, and the water slugs split into multiple smaller water slugs before reaching the condenser region. The water slugs then agglomerate in the condenser region due to the sudden reduction in temperature, and the pressure of the system is reduced due to the sudden phase shift. Due to this pressure change, the water plugs move downwards towards the evaporator region (t = 0.48s). The static phase is present for a shorter duration before oscillating again (t = 0.60s to t = 0.78s); the vapour plugs pulsate in the same position during this static phase and do not move from one region to another. The static phase reoccurs for 0.2s (t = 1s to 1.2s). The water slugs oscillate about the mean position.

**Fig 8:**
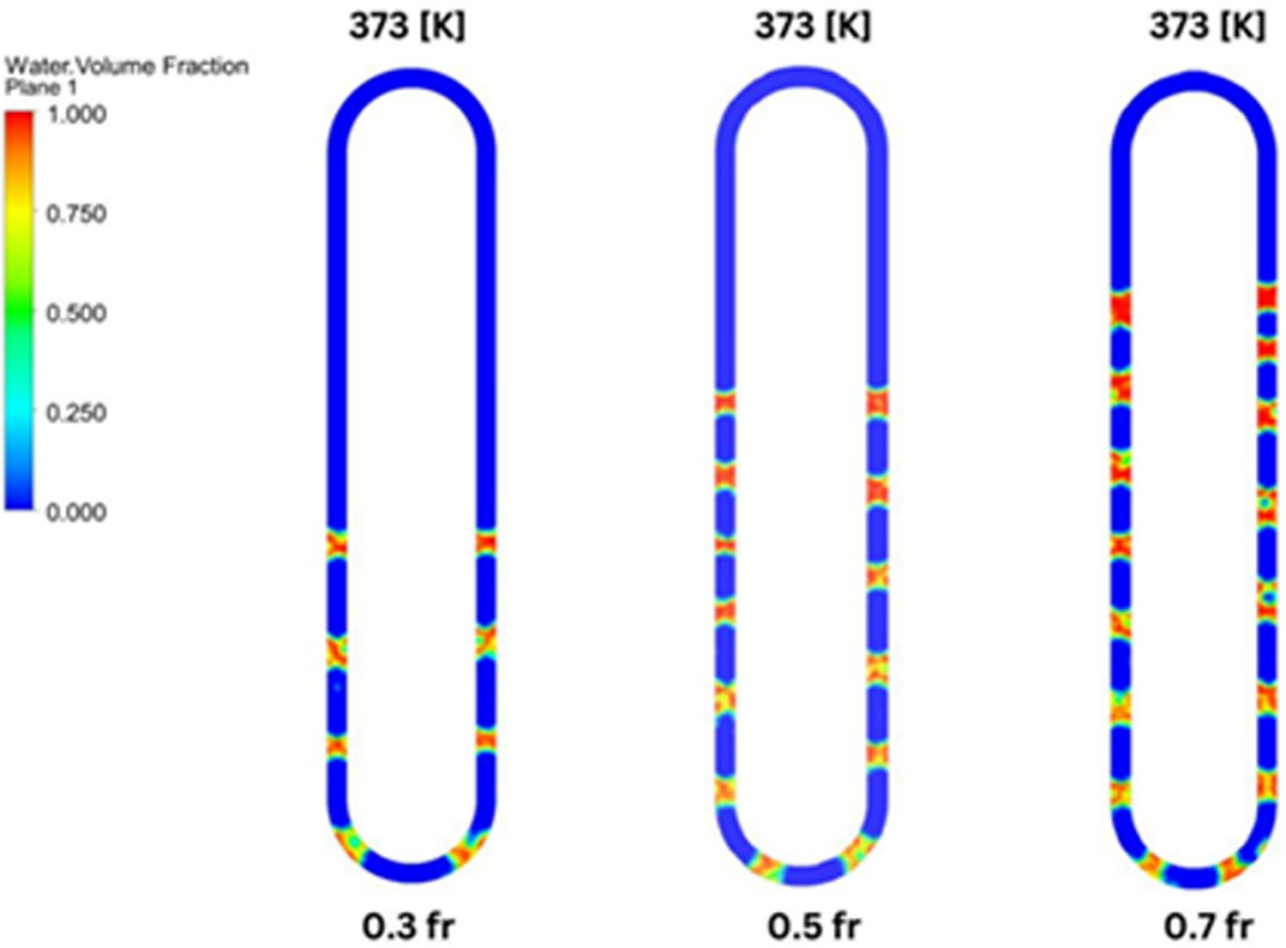
VOF contour for cases 1, 4, 7 at t=0.3s.

**Fig 8** shows that in the 0.7 fractions (case 1), the fluid movement is earlier than the 0.5fr (case 4) and does not complete the cycle, thus removing lesser energy. For the 0.3 filling ratio cases, it was found that there was an inordinate delay in the rise of slugs, which also effectively had a lower removal of energy despite higher heat flux. Therefore, input heating power significantly affects the evaporator’s and condenser’s thermal resistance. In addition, the mechanism of movement is also due to factors such as capillary force, filling ratio, and fluid-thermo physical properties. The following section discusses the effect of capillary forces and pressure differences due to the filling ratio.

### 4.2 Force variations acting on slugs for higher filling ratio

**Fig 9:**
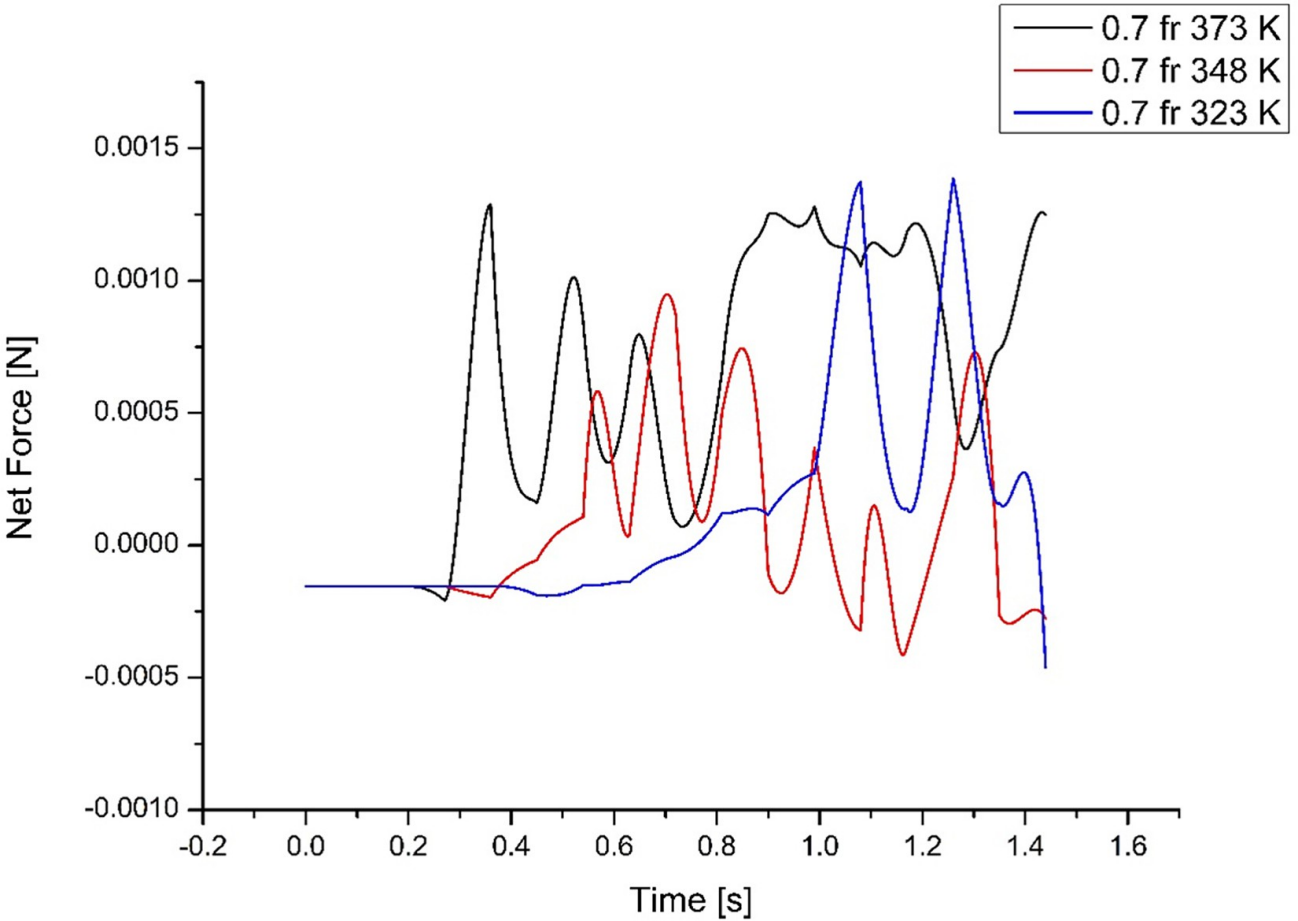
Force variations acting on slugs for higher filling ratio (case 1, 2 & 3).

The higher filling ratio has a higher mass in the evaporator region, and the wall temperature creates a force that must be sufficient to create a driving force upwards. The greater the temperature, the higher the driving force. In **Fig 9**, the thermodynamic force overcomes the gravitational force at 0.3s for the highest temperature and 0.5s for case 2, and case 3 reaches the threshold force at 1s. This demonstrates that thermodynamic variables impact the movement of slugs more than the hydrodynamic forces. Case 1 has the highest filling ratio and highest temperature, thus reaching the minimum force required to move the slug upwards at the earliest. The decomposition of the net forces into capillary force, gravitational force, surface tension, pressure and thermodynamic forces is required. The condenser effect of temperature on individual filling ratios is analyzed.

**Fig 10:**
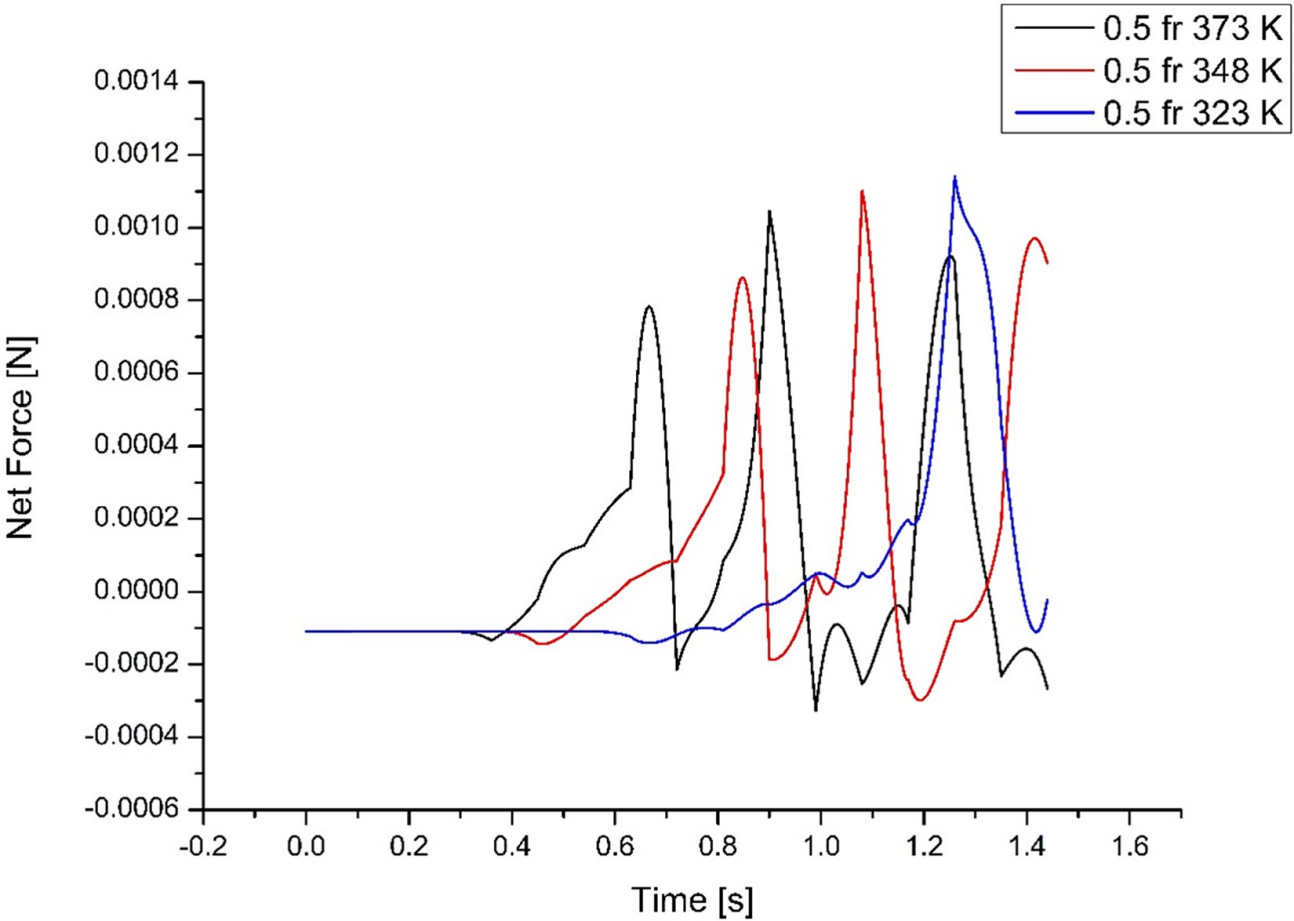
Force variations acting on slugs for medium filling ratio (case 4, 5 & 6).

**Fig 11:**
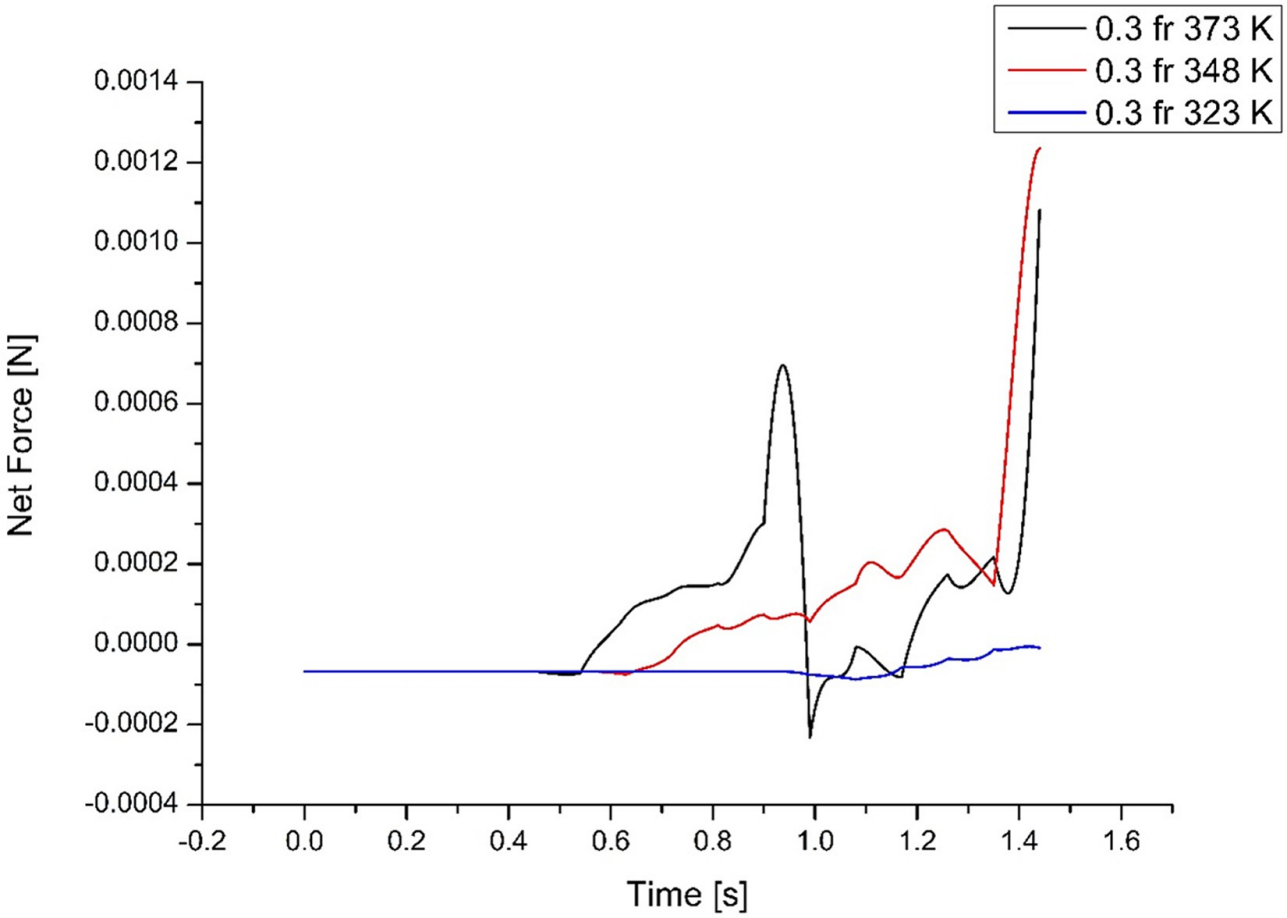
Force variations acting on slugs for low filling ratio (case 7, 8 & 9).

As explained earlier, a higher evaporator temperature has a faster response. For efficient transfer of energy, the evaporation and condensation time, as well as slug movement, must match. At high temperature (373K), the faster response is found to be detrimental as the time available for condensation is not sufficient for completing the cycle. The higher force developed prevents the slug from returning to complete the cycle, causing a dry-out state. At 348K, the thermodynamic force is found to match the condensation time that can overcome the hydrodynamic forces to complete the cycle. In effect, the forces generated due to the boiling and movement of vapour slugs, overcoming the hydrodynamic forces, should match to complete the cycle from the evaporator and condenser sides. Similar observations can also be drawn from **Figs 10** and **11** for medium and low-filling ratios.

The important factor for efficient heat transfer is the number of cycles that can be completed, not the mass of the fluid, although it can carry more heat. It is seen from **Figs 9–11** that the higher the mass, though, transfers energy with a quicker response, the delay in condensation reduces the number of heat transfer cycles. Similarly, a much lower mass cannot transfer enough heat, with poor response affecting the heat transfer cycle. Thus, the medium filling ratio in **Fig 10** illustrates the best performance by completing more heat transfer cycles.

**Fig 9** represents the variation in force for the three different evaporator temperatures. The buoyant force between the front and back of the slugs allows the movement of slugs upwards towards the condenser; an opposing force causes a downward movement. It is also observed that the force fluctuates in the positive direction. During this phase, the slugs do not complete the cycle but oscillate within the region, leading to the agglomeration of slugs and reduced thermal efficiency.

The net peak force acting on slugs is 1.39×10^-3^ N at t = 1.1s for case 1, 0.9×10^-3^ N at (t = 0.7s) for case 2, and 1.3×10^-3^ N at (t = 0.3s) for case 3. It can be observed that the movement of slugs depends on the input energy. The peak force is attained when the slugs are pushed towards the condenser before reaching stability. It can also be observed that the slugs take longer to reach the condenser and even longer to move towards the evaporator for the low temperature (323 K) for case 3. On the contrary, the force plot maintains a constant positive value for a longer duration for the high-temperature case, causing a dry-out condition. For case 2 (348 K) has the maximum number of oscillations for the given period, reaching the evaporator earlier than case 1. This delayed completion in the cycle is ineffective in evacuating the heat from the evaporator. Thus, this higher filling ratio case is more conducive.

### 4.3 Force variations acting on slugs for medium filling ratio

The fifty percent filling ratio cases are analyzed from the force plot presented in **Fig 10**. As was seen in the earlier higher filling ratio cases, the movement of slugs depends on input heat having a shorter duration for higher evaporator temperature. The peak force is also lower than in the previous case, and this also ensures force fluctuation towards positive and negative directions, helping the slugs to complete the cycle more times within the same time duration. Thus, the energy transfer is better and more efficient than in the earlier case.

The gradual increase in reaching the thermodynamic force to move the slugs could match the rate of condensation, thus increasing the number of heat transfer cycles. To achieve maximum efficiency the rate of evaporation and condensation must be similar, preventing dry-out. The net peak force acting on slugs is 1.12×10^-3^ N at t = 0.51s for case 4, 1.18×10^-3^ N at t = 0.84s for case 5, and 1.16×10^-3^ N at t = 1.32s for case 6.

### 4.4 Force variations acting on slugs for low filling ratio

From **Fig 11**, it can be seen that the force is not only lower but also delayed, which prevents the movement of slugs to the condenser, thereby reducing the amount of energy transferred in a given time. Only the case with higher temperatures provides us with the benefit of energy transfer by completing the cycle. Though the lower filling ratio has the most significant heat flux, the net energy transferred is the lowest. The net peak force acting on slugs is 1.08×10^-3^ N at t = 1.44s for case 7, 1.24×10^-3^ N at t = 1.44s for case 8, and 5.10×10^-6^ N at t = 1.47s for case 9. The higher filling ratio causes dry-out, and the lower filling ratio has no movement of slugs. Hence, the 0.5 filling ratio is the most efficient among the cases considered. Therefore, studying forces acting on the slugs for various filling ratios and geometric conditions would enable one to design and optimize the PHP to ensure more cycles within a period, thus helping faster heat evacuation from the target.

### 4.5 Regression analysis

The energy transfer can be computed within ± 30% using a set of non-dimension numbers [4]. As the design constraint would be temperature difference and filling ratio, a regression analysis is attempted to estimate an empirical equation as a function of Δ*T* and filling ratio. Since the non-dimensionless numbers vary based on the temperature differences in the CLPHP and the flow instabilities are dependent on the filling ratio, the empirical formula as a function of Δ*T* and filling ratio (*γ*) alone was sufficient for the given fluid. The Regression model is used to determine the relationship between the independent variable and the dependent variable. The equation below is used to find the best fit for a parabolic curve.

$$y=a_{1}x^{2}+a_{2}x+b$$ (20)

Where *a*_2_, *a*_1_, *and b* are coefficients of the quadratic equation. This quadratic equation was formulated by maximizing the coefficient of determination (*R*^2^). The simulated results are used to calculate Q for temperature gradients of 27 K to 77 K. (Δ*T*) considering it as an independent variable, and Q is considered the dependent variable by keeping the filling ratio constant. At the evaporator zone temperature of 373 K, when the filling ratio is increased by 40% from 0.5 to 0.7, the energy removed increased by 22.648% from 5.893W to 7.228W. The heat transfer rate (q) was obtained using Eqs 21 and 22 [40],

$$q=k_{eff}\frac{T_{w-}T_{ad}}{\Delta d}$$ (21)

$$\dot{Q}=\int_{i=1}^{n}\frac{Q_{i-1}{+[q_{evap}*A_{evap}+q_{cond}*A_{cond}]}_{i}}{n}$$ (22)

**Table 3** summarizes the $\dot{Q}$, R, and h for various fractions at different temperature differences [14, 41]. The first column of the table is used to develop the regression model. It can be seen from the second column that the thermal resistance decreases with decreasing temperature and increases with a decrease in the filling ratio. The exact opposite behaviour can be seen for the heat transfer coefficient (column 3). The heat removed depended on the completion of a cycle and was seen to be a quadratic behaviour.

**Table 3:** CFD results of the $\dot{Q}$, R, and h for various fractions at different temperature differences.

Case	Q [W]	R [K/W]	h [W/m^2^.K]
**Case 1**	5.5567	13.8571	177.9
**Case 2**	6.033	8.61926	286.008
**Case 3**	7.2284	3.73527	659.974
**Case 4**	3.1677	24.3079	101.415
**Case 5**	4.4702	11.6326	211.92
**Case 6**	5.8936	4.58124	538.103
**Case 7**	2.1961	35.0622	70.3088
**Case 8**	3.0484	17.0581	144.516
**Case 9**	3.2021	8.43197	292.361

Hence, the heat removed for a given filling ratio is represented as a quadratic equation as a function of Δ*T*, presented in Eq 23. The generic representation in matrix form is given in Eq 24. The polynomial constants are then estimated to yield an R^2^ value greater than 0.99, as per Eq 25.


$$\dot{Q}=a_{1}{\left(\Delta T\right)}^{2}+a_{2}(\Delta T)+b$$ (23)

$$\left[\begin{array}{ccc} \sum {\left(\Delta T\right)}_{i}^{4} & \sum ({\Delta T)}_{i}^{3} & \sum {\left(\Delta T\right)}_{i}^{2}\\ \sum ({\Delta T)}_{i}^{3} & \sum {\left(\Delta T\right)}_{i}^{2} & \sum {\left(\Delta T\right)}_{i}\\ \sum {\left(\Delta T\right)}_{i}^{2} & \sum {\left(\Delta T\right)}_{i} & n \end{array}\right]\left[\begin{array}{c} a_{1}\\ a_{2}\\ b \end{array}\right]=\left[\begin{array}{c} \sum {\left(\Delta T\right)}_{i}^{2}\dot{Q}\\ \sum {\left(\Delta T\right)}_{i}\dot{Q}\\ \sum {\dot{Q}}_{i} \end{array}\right]$$ (24)

$$R^{2}=1-\frac{\sum {\left({\dot{Q}}_{i}-a_{1}({\Delta T)}_{i}^{2}-a_{2}{\left(\Delta T\right)}_{i}-b\right)}^{2}}{{\sum ({\dot{Q}}_{i}-\bar{Q})}^{2}}$$ (25)

**Table 4** presents the various coefficients and the equations generated for the filling ratio (*γ*) 0.3, 0.5, and 0.7, as given in Eqs 26–28.

**Table 4:** Summation values for regression analysis.

Filling ratio	i=1	i=2	i=3	$\sum {\dot{Q}}_{i}$	$\sum {\left(\Delta T\right)}_{i}\;{\dot{Q}}_{i}$	$\sum {\left(\Delta T\right)}_{i}^{2}\;{\dot{Q}}_{i}$
**0.3**	3.22	3.0484	2.1961	8.4645	1320.46	205992
**0.5**	5.8936	4.4702	3.1677	13.5315	2110.91	329303
**0.7**	7.2284	6.033	5.5567	18.8181	2935.62	457957

$$\dot{Q}=0.000341{\left(\Delta T\right)}^{2}+0.024174(\Delta T)-0.303722,\gamma =0.3$$ (26)

$$\dot{Q}=-0.000290{\left(\Delta T\right)}^{2}+0.105688(\Delta T)-1.143528,\gamma =0.5$$ (27)

$$\dot{Q}=0.000211{\left(\Delta T\right)}^{2}+0.103130(\Delta T)-0.119448,\gamma =0.7$$ (28)

The above equations are further used to develop an empirical equation that is a function of both Δ*T* and *γ*. Eq 29 is again solved for coefficients, and the same is tabulated in **Table 5**.

**Table 5:** Summation values for empirical formula.

Filling ratio	i=1	i=2	i=3	$\sum {\dot{Q}}_{i}$	$\sum \gamma_{i}\;{\dot{Q}}_{i}$	$\sum \gamma_{i}^{2}\;{\dot{Q}}_{i}$
**Coefficient of** $\gamma_{i}^{2}$	-0.0006	0.038	2.5834	2.6208	3.9312	5.8968
**Coefficient of** *γ*_*i*_	0.0001	-0.0646	7.5667	7.5022	11.2533	16.87995
**constant**	0.0006	-0.0933	9.3271	9.2344	13.8516	20.7774


$$\dot{Q}=c\gamma^{2}+c\gamma +d$$ (29)

$$\left[\begin{array}{ccc} \sum \gamma_{i}^{4} & \sum \gamma_{i}^{3} & \sum \gamma_{i}^{2}\\ \sum \gamma_{i}^{3} & \sum \gamma_{i}^{2} & \sum \gamma_{i}\\ \sum \gamma_{i}^{2} & \sum \gamma_{i} & n \end{array}\right]\left[\begin{array}{c} c_{1}\\ c_{2}\\ d \end{array}\right]=\left[\begin{array}{c} \sum \gamma_{i}^{2}{\dot{Q}}_{i}\\ \sum \gamma_{i}{\dot{Q}}_{i}\\ \sum {\dot{Q}}_{i} \end{array}\right]$$

The final equation is presented in Eq 32 for the following limits: 0.3 <*γ*< 0.7 and 27<(Δ*T*)< 77. Eq 13, presented in Khandekar [4] to estimate the flux for a 50% filling ratio, is reproduced here as Eq 30.


$${\dot{q}}_{\left[4\right]}=0.54{Ka}^{0.47}{Ja}^{1.43}{Pr}^{0.27}N^{-0.27}{\left(\exp\left(\beta \right)\right)}^{0.48}\;W/m^{2}$$ (30)

Based on the above equation, the heat transfer rate for all filling ratios is modified and presented in Eq 31.


$$\dot{Q}={\dot{q}}_{\left[4\right]}.A_{CLPHP}.\gamma$$ (31)

$${\dot{Q}}_{reg}={\lambda_{1}.(\gamma )}^{2}+\lambda_{2}.(\gamma )+\lambda_{3}$$ (32)

$$\lambda_{1}=(-0.0025{\left(\Delta T\right)}^{2}+0.0055(\Delta T)-0.0020)$$ (33)

$$\lambda_{2}=(0.9237{\left(\Delta T\right)}^{2}-1.2520(\Delta T)+0.3305)$$ (34)

$$\lambda_{3}=(-40.2862{\left(\Delta T\right)}^{2}+57.1455(\Delta T)-10.9345)$$ (35)

**Fig 12:**
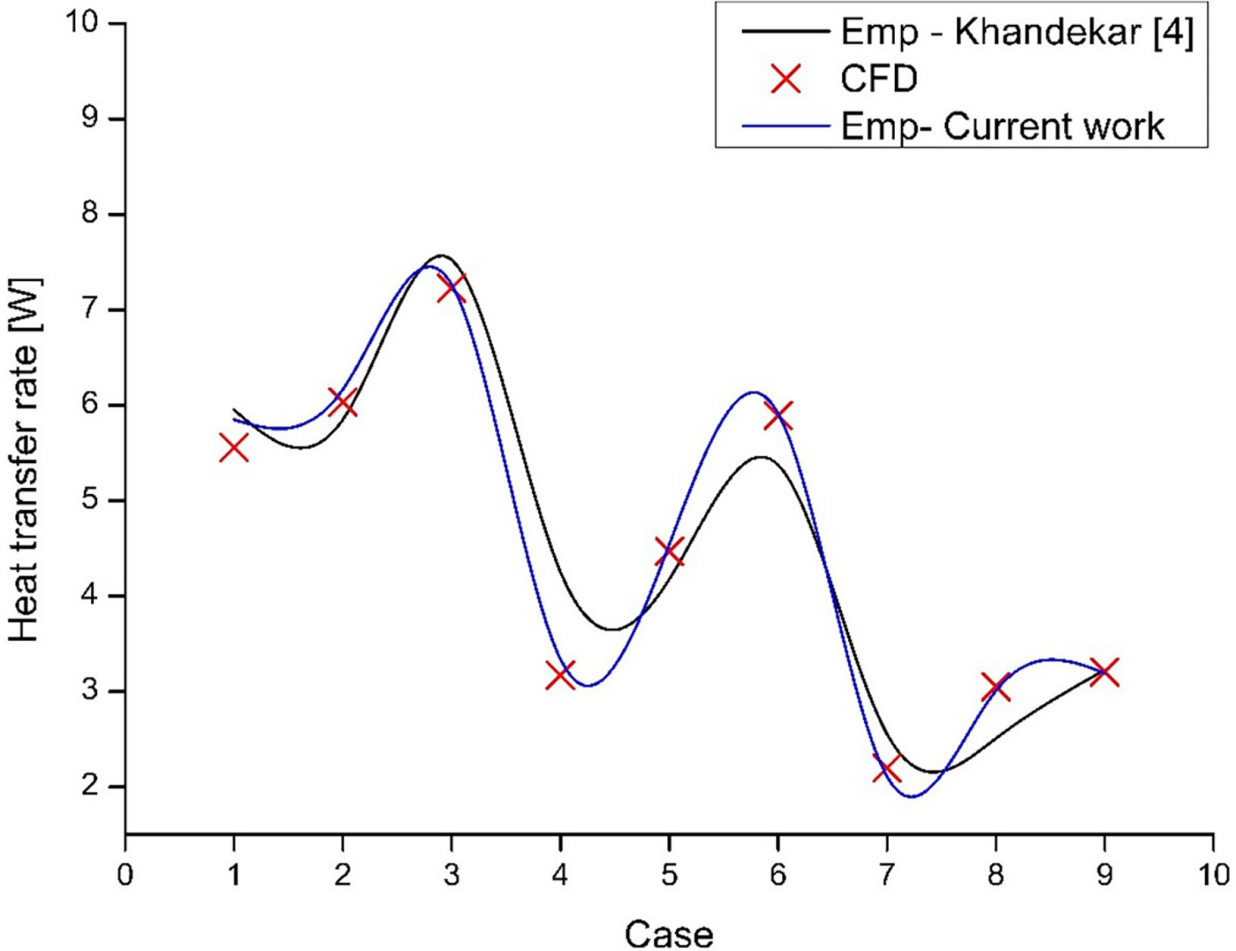
Heat transfer rate empirical model comparison.

The empirical results obtained from the present study (Eq 32) and that from the set of non-dimensionless numbers (Eq 30), along with the results obtained using CFD for the 9 cases, are presented in **Fig 12**. It was mentioned that the Empirical equation of [4] matches within ± 30%. Here, it can be seen to match quite well. The present empirical equation as a function of Δ*T* and the filling ratio is sufficient to represent the heat transfer rate. The reason is that the non-dimensionless numbers used in Eq 30 are dependent only on the mean temperature of the evaporator and condenser, irrespective of the filling ratio. Hence, a regression model based on Δ*T* and *γ* alone is sufficient. However, this equation will not be valid for other working fluids, and a separate empirical relation needs to be developed.

## 5. Conclusions

This study investigated the force variation acting on the slugs by considering the thermodynamic and hydrodynamic forces. The numerical model was validated against theoretical and experimental correlation. Three filling ratios (0.3, 0.5, and 0.7) and evaporator temperatures (323K, 348K, and 373K) were used to predict the net force acting on the slugs. A semi-empirical equation was developed to estimate the heat transfer rate, and an equation was developed to assess the pressure difference in the slugs. The key findings of the study are as follows:

- The medium filling ratio with 373K evaporator temperature was the most efficient, considering the premature dry-out condition in a high filling ratio and the delayed formation of slugs in a low filling ratio. It was also found that higher temperature creates greater force acting on the slug.
- The filling ratio impacts the thermal efficiency more than the temperature difference between the evaporator and condenser. It was seen that the 0.5 filling ratio performed better than all the other cases.
- 0.5 filling ratio was found to be the most effective in heat removal capacity over a more extended period due to the maximum number of cycles. The oscillations are caused by the increasing randomness, leading to increased saturation temperature and the variation of the forces acting on slugs.
- Completing the thermal cycle and oscillations/ instabilities varies with filling ratio rather than temperature. Heating power and movement of liquid slugs must be considered before designing a CLPHP.
- From the CFD studies, a semi-empirical formulation as a function of Δ*T* and *γ* was developed and found to match the empirical equation as a function of non-dimensionless numbers. Since the non-dimensionless numbers in Eq 30 vary based on the temperature differences in the CLPHP and the flow instabilities are dependent on the filling ratio, the empirical formula as a function of Δ*T* and *γ* alone was sufficient for the given fluid. While performing a physical experiment, the CLPHP is evacuated to the desired pressure and charged with a known quantity of working fluid. However, the liquid does not remain in the evaporator zone and disperses itself randomly. While performing CFD studies the fluid is confined to the evaporator section before the start of the simulation. This will have an effect during the initial loop and subsequently match with experimental results. This limitation needs to be acknowledged when comparing CFD studies with experiments.

The semi-empirical equations presented here is only valid for water. Future research is required to perform similar simulations for binary fluids (reduces the forces due to hydrodynamic variables), fluids with additives, and nanoparticles (increases the forces due to thermodynamic variables) and generate an empirical solution as presented above. The decomposition of the net force into individual thermal and hydrodynamic forces is important for understanding the complex thermo-hydrodynamic coupling.

## Abbreviations

A: Area (m^2^)
D: Diameter of the CLPHP (m
N: Number of turns
$\dot{Q}$: Heat transfer rate (W)
*T*: Temperature (K)
G: Acceleration due to gravity (m/s^2^)
*l*: Length (m)
*h*: Specific heat (kJ/kg)
$\dot{q}$: Heat flux (W/m^2^)
*r*: Radius of CLPHP (m)
**Greek symbol** *α*: Contact angle (deg
*γ*: Filling ratio
*θ*: Inclination angle (deg
*μ*: Dynamic viscosity (Pa.s)
*ρ*: Density (Kg/m^3^
*σ*: Surface tension (N/m
**Subscripts** *w*: Wal
*ad*: Adiabatic
*cap*: Capillary
*cond*: Condenser
*eff*: Effective
*evap*: Evaporato
*l*: Liquid
*p*: Pore
*push*: Pushing
*v*: Vapou
**Dimensionless numbers** Bo: Bond number
*Ka*: Karman Number
*Ja*: Jakob Number
*Pr*: Prandtl Number
